# Genes Potentially Associated with Familial Hypercholesterolemia

**DOI:** 10.3390/biom9120807

**Published:** 2019-11-29

**Authors:** Svetlana Mikhailova, Dinara Ivanoshchuk, Olga Timoshchenko, Elena Shakhtshneider

**Affiliations:** 1 Federal research center Institute of Cytology and Genetics, SB RAS, 630090 Novosibirsk, Russia; mikhail@bionet.nsc.ru (S.M.); dinara2084@mail.ru (D.I.); lentis@yandex.ru (O.T.); 2 Institute of Internal and Preventive Medicine—branch of Institute of Cytology and Genetics, Siberian Branch of Russian Academy of Sciences (SB RAS), 630004 Novosibirsk, Russia

**Keywords:** Familial hypercholesterolemia, *STAP1*, *CYP7A1*, *LIPA*, *ABCG5*, *ABCG8*, *PNPLA5*

## Abstract

This review addresses the contribution of some genes to the phenotype of familial hypercholesterolemia. At present, it is known that the pathogenesis of this disease involves not only a pathological variant of low-density lipoprotein receptor and its ligands (apolipoprotein B, proprotein convertase subtilisin/kexin type 9 or low-density lipoprotein receptor adaptor protein 1), but also lipids, including sphingolipids, fatty acids, and sterols. The genetic cause of familial hypercholesterolemia is unknown in 20%–40% of the cases. The genes *STAP1* (signal transducing adaptor family member 1), *CYP7A1* (cytochrome P450 family 7 subfamily A member 1), *LIPA* (lipase A, lysosomal acid type), *ABCG5* (ATP binding cassette subfamily G member 5), *ABCG8* (ATP binding cassette subfamily G member 8), and *PNPLA5* (patatin like phospholipase domain containing 5), which can cause aberrations of lipid metabolism, are being evaluated as new targets for the diagnosis and personalized management of familial hypercholesterolemia.

## 1. Introduction

Familial hypercholesterolemia (FH) is a monogenic disease with mostly autosomal dominant inheritance and is characterized by substantial elevation of the blood level of cholesterol associated with low-density lipoproteins (LDL-C) and by the early development and progression of atherosclerosis [1].

FH is the most prevalent congenital metabolic disorder [2]. In FH with childhood onset, there is significant elevation of total cholesterol and LDL-C accompanied by a normal or moderately elevated concentration of triglycerides in the blood [3,4]. FH poses a risk of early complications, such as ischemic heart disease (IHD), cerebral atherosclerosis, and arterial atherosclerosis of lower extremities. The average age of clinical manifestation of IHD is <45 years among males and <55 years among females [5], whereas in the homozygous form of FH, clinical IHD signs may present in the second decade of life [6]. Despite the high prevalence of this disease and availability of effective treatments, FH often goes undiagnosed and untreated, especially in children [7]. For a timely diagnosis of FH, some investigators have proposed the method of cascade genetic screening [8].

Cascade genetic screening of FH is a step-by-step identification of patients with FH. At the beginning, screening is performed to identify an elevated cholesterol level in a patient; next, for a patient with hypercholesterolemia, the family history and clinical manifestations should be analyzed. If a diagnosis of probable or definite FH is made according to the lipid-based criteria of the Simon Broom Register Group (UK) and the Dutch Lipid Clinic Network Criteria [9,10,11], then the patient is scheduled for a molecular genetic analysis. The next step is quantitation of blood lipids in all first-degree relatives of the proband. As recommended by the European Atherosclerosis Society, the screening of cholesterol levels in a population should include all people older than 20 years. If there is a family history of FH or early-onset IHD, the quantification of total cholesterol in blood serum should be started at the age of 2 years [12].

In case FH is confirmed genetically in the proband, subsequent genetic testing is recommended for the identification of affected family members [13]. As new patients with FH are identified, their relatives are examined too. The cascade screening is the most effective method for identifying people with FH [8].

This method has been officially and ethically approved by the World Health Organization. This disease poses a serious health hazard, while a diagnostic test is available, as are preventive measures.

**Figure 1:**
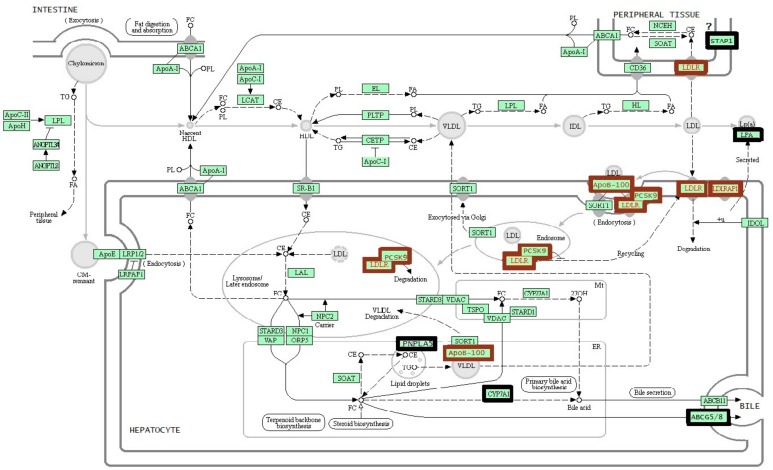
The scheme of cholesterol metabolism [14]. A brown frame indicates the genes recommended for standard genetic testing for familial hypercholesterolemia. A black frame indicates the genes that are discussed in this review. CE: cholesterol esters; CM: chylomicron; FA: fatty acids; FC: free cholesterol; HDL: high-density lipoproteins; IDL: intermediate-density lipoproteins; LDL: low-density lipoproteins; PL: phospholipid; TG: triglycerides; VLDL: very-low-density lipoproteins.

*LDLR* (low-density lipoprotein receptor), *APOB* (apolipoprotein B), and *PCSK9* (proprotein convertase subtilisin/kexin type 9) are the genes whose mutations determine the development of the autosomal dominant form of FH, and *LDLRAP1* (low-density lipoprotein receptor adaptor protein 1) is a gene associated with the autosomal recessive form of the disease (Figure 1) [14]. Mutations in *LDLR* are detectable in 80%–85% of FH cases; when a molecular genetic cause of FH is known, mutations of *APOB* are found in 5%–7% of the patients; mutations in the *PCSK9* gene are detectable in fewer than 5% of the cases, and mutations of *LDLRAP1* occur in <1% of the cases [7,9,15,16,17].

In this review, we discuss six genes *STAP1* (signal transducing adaptor family member 1), *CYP7A1* (cytochrome P450 family 7 subfamily A member 1), *LIPA* (lipase A, lysosomal acid type), *ABCG5* (ATP binding cassette subfamily G member 5), *ABCG8* (ATP binding cassette subfamily G member 8), and *PNPLA5* (patatin like phospholipase domain containing 5) that are often mentioned lately in association with FH (Figure 1).

A negative result of the genetic screening for *LDLR*, *APOB*, *PCSK9*, and *LDLRAP1* mutations does not rule out FH. In 20%–40% of the cases of FH, the molecular genetic testing does not detect changes in the above genes [18]. In some FH cases, an elevated concentration of LDL-C may be inherited polygenically [19]. The average speed of discovery of new genes associated with FH has been approximately one gene per decade since the 1970s [20].

This review discusses the genes described recently as potentially associated with the formation of the FH phenotype (*STAP1*, *CYP7A1*, *LIPA*, *ABCG5*, *ABCG8*, and *PNPLA5*).

## 2. *STAP1*

The STAP1 protein (signal-transducing adaptor protein family member 1), also known as BRDG1 (B-cell antigen receptor downstream signaling 1), was discovered in immune cells. Its highest expression has been documented in the appendix, lymph nodes, and spleen. The STAP1 protein is encoded by *STAP1*, which is located in chromosomal region 4q13.2 and contains 10 exons (https://www.ncbi.nlm.nih.gov/gene/26228). The STAP1 protein, depending on alternative mRNA splicing, consists of 295 or 314 amino acid residues (aa) and contains several phosphorylation sites and an N-terminal proline-rich region (Pro6–Pro11), followed by pleckstrin homology (PH: Leu24–Thr152) and Src homology 2 (SH2: Asn173–Cys269) domains. The PH domain is responsible for the interaction of this protein with certain phospholipids, thus enabling its anchoring in a cellular membrane. SH2 domains mediate the binding of this protein to phospho-tyrosine-containing sequences, thereby allowing for protein–protein interactions [21,22,23,24].

STAP1 is associated with protein kinase Tec, which is activated by surface receptors of B cells, CD19, and CD38 and is phosphorylated by this kinase. The overexpression of STAP1 increases B-cell receptor-mediated activation of the CREB (cAMP response element-binding) protein. It is believed that STAP1 implements a positive feedback loop, thus increasing the activity of Tec tyrosine kinase. In mice, the activity of the ortholog of STAP1 depends on its isoform [21,23] and is a substrate of the murine receptor tyrosine kinase c-Kit, which is necessary for the differentiation of hematopoietic stem cells [22]. It should be noted that the treatment of chronic myeloid leukemia with inhibitors of this tyrosine kinase elevates the plasma level of cholesterol [25,26]. STAP1 participates in the anti-inflammatory activation of glia, and in this way, possibly contributes to the apoptosis and degeneration of neurons [27].

During a study on the association between the expression of genes in leukocytes and the level of plasma lipids, it was demonstrated that *STAP1* is one of the genes of the humoral immune response and that *STAP1* expression positively correlates with the plasma concentration of lipids [28].

During genetic mapping of the members of five Dutch families with autosomal dominant hypercholesterolemia, a chromosome 4 region was found to be linked with this disease. As a result, the p.Glu97Asp mutation (rs779392825) was identified in the *STAP1* gene. One more carrier of p.Glu97Asp and three additional mutations, p.Leu69Ser (c.206T>C, rs938523789), p.Ile71Thr (c.212T>C, rs141647940), and p.Asp207Asn (c.619G>A, rs146545610), were identified in the coding regions of the *STAP1* gene in 400 unrelated probands with FH and without mutations in known FH-associated genes using a sequencing analysis. All the detected mutations were located in highly conserved loci [29].

In a sample of German patients with hypercholesterolemia, a new mutation in *STAP1*—c.139A>G (p.(T47A), rs793888522)—was found to be co-segregated in the family of a patient with myocardial infarction [30].

During a screening of patients with elevated LDL-C in Spain, a mutation (c.291G>C, p.Glu97Asp) in the *STAP1* gene was found in the family of one patient [31]. A heterozygous mutation, rs199787258 in the *STAP1* gene (c.526C>T, p.Pro176Ser), was also discovered in a patient with dyslipidemia and in his relatives in Spain. A bioinformatic analysis of the sequence of the mutant STAP1 revealed that a substitution of nonpolar proline with hydroxyl-containing serine completely altered the SH2 domain’s structure in this protein [32].

In a study of patients aged up to 35 years with FH in China, one of them was found to carry a novel missense mutation, c.596A>G p.Asn199Ser, in the *STAP1* gene [33].

Patients with mutations in *STAP1* have a less pronounced pathological phenotype as compared to the patients who carry mutations in *APOB* or *LDLR*. Meanwhile, carriers of mutations in *STAP1* are characterized by a significantly higher level of triglycerides in comparison with the carriers of mutations in the above genes [28,29]. It has been reported that some pathogenic mutations—Pro176Ser, Leu69Ser, and Asp207Asn—have incomplete penetrance, suggestive of polygenic inheritance of dyslipidemia [29,32].

On the contrary, M. Hartgers et al. (2019) demonstrated that the p.Leu69Ser, p.Ile71Thr, and p.Glu97Asp variants of STAP1 are not associated with an elevated LDL-C level and do not affect LDL-C homeostasis in the liver [34]. The absence of a statistically significant association of rs199787258 (p.Pro176Ser) of *STAP1* with blood lipid levels was demonstrated in a study by M. Danyel et al. (2019) [35]. These data show that substitutions in the *STAP1* gene most likely are not the cause of definite FH, and this gene’s role in the regulation of lipid metabolism requires further research.

## 3. *CYP7A1*

The *CYP7A1* gene is located in the 8q12.1 chromosomal region, contains six exons, and the encoded protein consists of 504 aa (https://www.genecards.org/cgi-bin/carddisp.pl?gene=CYP7A1). In the region between enhancers in the 5′ untranslated region (UTR) of *CYP7A1* and its intron 2, a haplotype block approximately 7000-bp long was discovered that covers the binding sites of numerous transcription factors—NR1H4 (nuclear receptor subfamily 1 group H member 4), NR1H2 (nuclear receptor subfamily 1 group H member 2), NR1I2 (nuclear receptor subfamily 1 group I member 2), NR0B2 (nuclear receptor subfamily 0 group B member 2), FGF19 (fibroblast growth factor 19), HNF4A (hepatocyte nuclear factor 4 alpha) and NR5A2 (nuclear receptor subfamily 5 group A member 2)—which regulate *CYP7A1* expression [36].

*CYP7A1* encodes protein 1, subfamily A, family 7 of cytochrome P450; CYP7A1 is synthesized in liver cells and functions as cholesterol 7-α monooxygenase. This enzyme catalyzes the first step of the main catabolic pathway of cholesterol in the human body: the transformation of cholesterol into bile acids via attachment of a hydroxyl group at position 7-α. Bile acids, although necessary for lipid assimilation, are potentially toxic to cells in the human body because these acids are detergents. Their concentrations are tightly regulated, for example, in accordance with the feedback loop principle [37]. A large number of factors determining CYP7A1 activity have been identified in the liver. The transcription of *CYP7A1* is stimulated by glucose via epigenetic regulation of histone acetylation [38]. In HepG2 cells, negative regulation of *CYP7A1* expression by microRNA-17 has been proven [39].

There are several polymorphic variants in *CYP7A1* that are associated with total cholesterol and LDL-C levels as well as with the predisposition to cholesterol metabolism-related diseases. The variant rs10957057 (in the 3′ UTR of *CYP7A1*) is associated with the levels of total cholesterol and LDL-C among Caribbean Hispanics [40]. The rs7833904 variant, which is located in the 5′ UTR of *CYP7A1*, modifies the risk of IHD, especially in males [41]. The variants rs72647413 (p.Thr193Ile) and rs139396617 (Arg260Leu) are associated with the level of LDL-C [42]. The rs3808607G variant at position −203 in the promoter and rs2081687 in the 3′ region of the gene correlate with elevated levels of total cholesterol and LDL-C, and the effects of these polymorphisms on the two indicators are opposite [42]. Carriers of the rs3808607T allele manifest changes in the diurnal activity of *CYP7A1*, whereas in carriers of the rs3808607G allele, there are no diurnal changes [43]. The single-nucleotide polymorphism (SNP) rs9297994 in the 3′ UTR of *CYP7A1* is also in linkage disequilibrium with rs3808607 in the promoter region. It has been demonstrated that these two SNPs have opposite effects on the mRNA expression of *CYP7A1* and determine the risk of IHD and diabetes mellitus [44].

## 4. *LIPA*

This gene encodes a lysosomal acid lipase (lipase A, hydrolase of cholesterol esters). The gene is located in chromosomal region 10q23.31 within a cluster of acid lipase genes and contains 10 exons [45]. *LIPA* is expressed in the spleen, small intestine, lymph nodes, liver, and lungs (https://www.ncbi.nlm.nih.gov/gene/3988). The encoded polypeptide can be 283 or 399 aa long, depending on the length of the N terminus of an RNA splice variant, and contains a 21 aa leader peptide necessary for the transport of this protein into lysosomes [45].

Lipase A participates in the hydrolysis of triglycerides and complex esters of cholesterol, which are delivered to lysosomes via receptor-mediated endocytosis of LDL particles, thereby providing cholesterol for cell growth and membrane function. Lipase A deficiency is inherited in an autosomal recessive manner. Depending on zygosity and the degree of protein damage, variants in this gene can cause Wolman disease, which develops in the first months after birth, or cholesterol ester storage disease, which manifests later, in childhood or adulthood [46,47,48,49]. These diseases are characterized by intracellular storage of cholesterol esters and disturbances in the control of cholesterol synthesis. Phenotypic variation differs considerably among patients [49,50,51], and the most prevalent symptoms of lipase A deficiency are hepatomegaly and a characteristic lipid profile of blood serum: high levels of total cholesterol, LDL-C, and triglycerides and a low concentration of high-density lipoprotein cholesterol (HDL-C). Substitutions in *LIPA* are also associated with IHD, metabolic complications of obesity [52], and FH [49,53].

The active catalytic site of this lipase consists of 3 aa (Ser174, Asp345, and His374), whereas cysteine residues Cys248/Cys257 form a disulfide bridge. The site of mannose-6-phosphate N-glycosylation (aa 161–163: Asn-Lys-Thr) and a C-terminal motif (aa 396–397: Arg–Lys) as well as the two most probable sites of N-glycosylation of LIPA (Asn36-Val37-Ser38 and Asn273-274Met-275Ser) are conserved among vertebrates [54].

The binding sites for the transcription factors NKX2-5 (NK2 homeobox 5), COMP1 (muscle specific transcription enhancer), HNF3B (hepatocyte nuclear factor 3-beta), GFI1 (zinc finger protein GFI1), RORA2 (alpha orphan nuclear receptor), EVI1 (zinc finger protein EVI1), FREAC4 (forkhead box protein), STAT3 (identified in the promoters of acute-phase genes), HEN1 (helix-loop-helix protein 1), and OCT1 (transcription factor that binds to the octomer motif) and CpG islands have been found in the 5′ UTR of *LIPA*, and a binding site for HNF4 (hepatocyte nuclear factor 4) was identified in the 3′ UTR [54].

One of the most prevalent *LIPA* SNPs that is known to affect the protein function is Thr16Pro (rs1051338) located in the leader peptide. This substitution reduces the amount of the enzyme without affecting its activity [53,55]. Thr16Pro frequency is 0.12 in Africans, 0.34 in Europeans, and up to 0.42 in the South Asian population. In patients with cholesterol ester storage disease, the most prevalent substitution (more than 50% of the cases) is Gln298Gln (894G>A, rs116928232), which disrupts a splice site and causes exon 8 skipping and a loss of 24 aa [46,47,56,57]. The frequency of its carriage in white populations is 1 per 200 to 1 per 1000 people, whereas the frequency of cholesterol ester storage disease—which results from the homozygosity of Gln298Gln—is ~1 per 130,000 people. It has not been found in Africans [56,57,58]. The residual enzymatic activity of mutated protein is 3%–8%. It is reported that among both males and females, heterozygous carriers of rs116928232 have a significantly elevated level of total cholesterol; furthermore, in males, there is elevated LDL-C concentration [59].

In addition, in the *LIPA* gene of patients with Wolman disease, cholesterol ester storage disease, or FH, many other rare rearrangements have been found, including a long deletion (parts of intron 3 and exon 4 [59,60]); frame shift mutations c.684delT (p.Phe228Leufs*13) [48], c.229+3A>C [51], and c.482delA [60]; and >50 missense and nonsense mutations as well as substitutions in the promoter region and splice sites with a predicted pathogenic effect [50,51,53,61,62,63,64,65].

A functional analysis of the substitutions in *LIPA* has been performed in vitro on HeLaT-Rex cells that were transfected with plasmids carrying *LIPA* variants with 41 missense mutations described in the Human Genome Mutation Database in 2016 (http://www.hgmd.cf.ac.uk/ac/index.php). In the transfected cells, the enzymatic activity was assessed; as a result, for 32 of the 41 analyzed substitutions, lipase A residual activity was less than 10%; these data indicate the pathogenicity of these mutations [53].

It is likely that heterozygous substitutions in *LIPA* can lead to lipase A deficiency and cause hypercholesterolemia [53,64,65,66]. In a study by J. Cebolla et al. (2019), among 24 patients with primary hypercholesterolemia or deficiency in lysosomal acid lipase, but without pathogenic mutations in *LDLR*, *APOB*, *PCSK9*, or *LDLRAP1*, there were patients with two known and one novel substitution in the *LIPA* gene. Exon 8 splice junction mutation E8SJM (NM_000235.2:c.894A>G (NP_001121077.1:p.Ser275_Gln298del)) was found in the homozygous state, in compound heterozygosity, and in combination with a previously unreported 25 bp deletion NM_000235.2:c.95+111_del25 (NP_001121077.1:p.Thr33*, located on the exon 1–intron 1 border). A novel single-nucleotide variant of *LIPA* (NM_000235.2:c.-106C>A) is located in the 5′ UTR in the heterozygous state [67].

## 5. *ABCG5* and *ABCG8*

These genes (ATP-binding cassette subfamily G, members 5 and 8) encode subunits of a membrane transporter of sterols. The two genes are located on opposite DNA strands head to head in the 2p21 chromosomal region; the distance between their start codons is 374 bp. Their shared promoter is located in this region, and contains regulatory sites for HNF4α (hepatocyte nuclear factor 4 alpha), NR5A2 (nuclear receptor subfamily 5 group A member 2), NFKB1 (nuclear factor kappa B subunit 1), and FOXO1 (forkhead box O1) [68,69]. The shared enhancer of the two genes was mapped to a site located 100,000 bp away from the promoter [70]. Meanwhile, the *ABCG5* gene overlaps in its 3′ end starting with exon 5 with the *DYNC2LT1* (dynein cytoplasmic 2 light intermediate chain 1) gene, whereas *ABCG8* also encodes an antisense transcript, LOC102725159, 402 bp long, which overlaps with a part of exon 3 and intron 2. Traditionally, this evolutionarily conserved locus is called the “sitosterolemia locus” because this region is linked to an autosomal recessive disease called sitosterolemia, characterized by accumulation of xenosterols, macrothrombocytopenia, and early-onset IHD [68,69,71]. β-Sitosterol is the most widespread phytosterol, whereas phytosterols are the most prevalent xenosterols in the human body; for this reason, instead of the term “sitosterolemia,” the words phytosterolemia and xenosterolemia are often used.

The *ABCG5* gene contains 15 exons (https://www.ncbi.nlm.nih.gov/gene/?term=ABCG5). A prevalent mRNA splice variant is encoded by 13 exons, and the protein consists of 651 aa [72]. *ABCG8* also contains 15 exons (https://www.ncbi.nlm.nih.gov/gene/?term=ABCG8), whereas a prevalent mRNA splice variant is encoded by 13 exons, and the resultant protein is 677 aa long [68].

Both proteins are composed of a magnesium-dependent ATP-binding domain at the N terminus (containing conserved peptide motifs Walker A and B) and a transmembrane domain including six transmembrane helices. The sixth transmembrane helix of ABCG8 carries a consensus sequence for the recognition of cholesterol; due to this sequence, only ABCG8 in the ABCG5/8 pair can serve as a sensor of the cholesterol level during its excretion [73].

The expression of both genes is most active in the liver and intestines and is present in the gallbladder. The heterodimeric protein complex ABCG5–ABCG8 is found in the apical membrane of enterocytes and in the channel-containing membrane of enterocytes [18].

The superfamily of ABC transporters includes proteins taking part in the transfer of various molecules across internal and external cell membranes. ABCG5–ABCG8 is the main carrier of xenosterols and a transporter of excess cholesterol into the intestinal lumen from enterocytes and into bile from hepatocytes [68,69]. Moreover, it has been found that the ABCG5–ABCG8 complex lowers the absorption of cholesterol from the intestinal lumen [74,75].

On the basis of ABCG5–ABCG8 crystal structure, amino acid residues have been predicted whose substitutions disrupt the transport of cholesterol out of the cells; these are Arg389, Arg419, Asn437, Ile523, Arg550, Cys600, Gln604, and Met622 in ABCG5 and Thr400, Arg405, Leu501, Arg543, Leu572, Gly574, Gly575, and Leu596 in ABCG8 [73,76].

In the Latin American population, common SNPs in the *ABCG8* gene, i.e., Asp19His (rs11887534) and Thr400Lys (rs4148217)—having the frequencies of 10% and of 35%, respectively—are associated with gallstone disease. It is known that these SNPs do not affect the expression of *ABCG8* [77,78]. Nevertheless, His/His rs11887534 homozygotes have 5% lower levels (in comparison with the Asp/Asp genotype) of total cholesterol and LDL-C [79]. It is believed that the Asp19His variant simultaneously has a protective effect against myocardial infarction and increases the risk of gallstone disease [79,80]. Besides, the His allele of rs11887534 is associated with bile duct cancer [81].

Among the patients with sitosterolemia, there are homozygotes and compound heterozygotes of substitutions Trp361X (rs137852987), Cys574Arg (rs137852988), Tyr658X (rs137852989), Arg263Gln (rs137852990), Leu596Arg, Arg412X (rs137852991), del547C, or c965-1G>C in ABCG8 or Arg408X (rs119479065), Gln251X (rs140111105), Arg446X (rs199689137), Pro231Thr, IVS10(-1)G>T (rs768019354), IVS1(-1)G>A, del1523C, IVS12(+1)G>A, or Arg419His (rs119479067) in *ABCG5* [82,83,84,85,86,87,88]. It has been demonstrated that in various ethnic groups, the level of sitosterol is higher in the carriers of Gly574Arg (rs137852988) or Met429Val (rs147194762) variant [89,90]. In Asp19His (rs11887534) carriers, the concentrations of plant sterols in blood plasma are lower [82]. The plasma level of cholesterol is lower in Val632Ala homozygotes (rs6544718, minor allele frequency 0.22 in Europeans) [82]. Among patients with FH, carriers of the Gln604Glu allele (rs6720173) of *ABCG5* are reported to have a lower concentration of triglycerides and very-low-density lipoprotein, whereas the presence of intron variant rs4131229A>G, rs4148189C>T, or rs4289236G>A of this gene has been found to be associated with higher levels of total cholesterol and triglycerides as well as a lower level of HDL-C only in smokers [91]. In Spanish patients with hypercholesterolemia, Asn578Ser (rs146534033), Gly288Cys (rs139264483), Arg198Glu (rs141828689), Gly269Arg (rs552803459), and Asn296Ser (rs552803459) in *ABCG5* and Gly512Arg (rs376069170) in the *ABCG8* gene have been detected [92].

Among compound heterozygotes D19H/T400K of the *ABCG8* gene (rs11887534 and rs4148217), there is a higher risk of cardiovascular diseases among patients with hypercholesterolemia [93]. Among Chinese patients with IHD, the frequency of homozygotes CC (Thr/Thr) of Thr400Lys (rs4148217) is significantly higher, and in combination with smoking, they have an elevated level of triglycerides in comparison with genotypes AC and AA [94]. In two ethnic groups of China (Han and Mulao), it has been found that substitution Thr400Lys (rs4148217) influences the levels of triglycerides and HDL-C only in females, and this correlation is ethno-specific [95]. In a study by Teupser et al. (2010), alleles of *ABCG8* that are associated with a lower concentration of phytosterols turned out to be associated with a lower risk of IHD as well. Thus, the presence of a rare allele of rs41360247 is protective, whereas the presence of a rare allele of rs4245791 is pathogenic in terms of IHD. Besides, rs41360247 was found to be in linkage disequilibrium with Asp19His (rs11887534), whereas rs41360247 with rs4952688 in this gene. The carriage of the AA genotype of *ABCG8* rs4952688 lowers the expression of ABCG8 and ABCG5 by 40% in the liver; the mechanism of this phenomenon remains unknown [96]. Among Hungarian patients with cardiovascular diseases, the frequency of carriers of genotype Tyr/Tyr of substitution Y54Cys (rs4148211) in the *ABCG8* gene is lower among males younger than 50 with myocardial infarction, whereas the level of cholesterol is lower in Tyr/Tyr carriers than in the carriers of genotypes Tyr/Cys and Cys/Cys in a control sample [97].

The associations of individual substitutions in genes *ABCG5* and *ABCG8* with different phenotypic characteristics of their carriers can significantly differ between ethnic groups because linkage disequilibrium has been detected at the 2p21 locus [82,96,97] and because the regulatory regions and transcripts of the four genes overlap.

## 6. *PNPLA5*

The *PNPLA5* gene (patatin-like phospholipase domain-containing 5) belongs to the family of patatin-like phospholipases consisting of nine genes and one pseudogene [98,99]. Patatin-like phospholipases play a key role in energy metabolism, the hydrolysis of triglycerides, and the regulation of adipocyte differentiation [99].

The *PNPLA5* gene is located in the 22q13.31 locus near another gene of this family (*PNPLA3*) and contains nine exons. *PNPLA5* mRNA has several splicing variants, with the maximal size of the resulting polypeptide of 429 aa.

PNPLA5 contains a conserved N-terminal adiponectin (patatin) domain (aa 12–181) [98] and a C-terminal domain, which varies among the members of the PNPLA family. Within the patatin domain of the proteins of this family, there are two conserved catalytic lipase centers: Gly-X-Ser-X-Gly and Asp-X-Gly/Ala [100]. The region “aa 340–364” of the PNPLA5 C-terminal domain is the so-called “basic patch.” Because of its negative charge, this region is responsible for the ability of PNPLA phospholipases to bind to lipid droplets in the cytoplasm [101]. It has been shown that the N terminus of PNPLA5 regulates the binding ability of the C terminus, because in vitro, the C terminus separately binds to membranes more effectively than the full-size protein does [101]. Within regions “aa 33–36” and “aa 353–356” of this protein, there are predicted binding sites for glycosaminoglycans [99].

*PNPLA5* expression is detectable in the brain, skin, and gallbladder [102], and modest expression has been registered in the liver [99] and adipose tissue [98]. The protein is detectable in the cytoplasm and is normally located on the surface of lipid droplets, which are conserved organelles composed of lipids in the form of triacylglycerides and sterol esters enclosed by a membrane monolayer [101]. The lipids found in these droplets probably serve as an energy depot of the cell and as membrane precursors for autophagosomes [103].

It has been demonstrated that tissue levels of *PNPLA5* mRNA are lower as compared with other lipases of this family [98,99]. The protein is detected on the surface of lipid droplets only in 35% of all cells. The expression of *PNPLA5* is regulated intracellularly depending on the physiological state of a specific cell type [101].

In vitro overexpression of *PNPLA5* lowers the level of intracellular triglycerides; therefore, it has been concluded that the main function of PNPLA5 is lipolysis. The *PNPLA5* mRNA level is lower in the adipose tissue of a mouse strain with genetically caused obesity (*ob*/*ob* mice) and in the adipose tissue of starving animals, but is higher in the liver of *ob*/*ob* mice [98]. In later publications, PNPLA5 has been suggested to transmit a signal triggering the formation of autophagosomal membranes. The lipid stores for their formation are mobilized from lipid droplets during autophagy. It is reported that the ability of lipid droplets to form autophagosomes increases depending on PNPLA5 [103].

During whole-exome analysis of African Americans and white Americans with extremely low or extremely high LDL-C levels, the genes whose rare and low-frequency variants occur significantly more frequently were identified in these groups. Aside from the known dyslipidemia-associated genes, rare (frequency <0.001), possibly pathogenic variants (causing aa substitutions or changes in splice sites) of the *PNPLA5* gene were found in both groups. The correlation of rare variants of *PNPLA5* with the LDL-C level was confirmed in an additional population. People of African and European origin have different sets of substitutions in this gene; most of the rare SNPs occur in the group with elevated LDL-C concentration. Among the variants found in the European-origin subjects with extremely high LDL-C levels, there were Cys68Phe (rs199891914), His87Asn (rs201608516), His95Gln (rs377273512), Ser199Ile (rs145976734), Phe222Leu (rs374016542), Ala317Val (rs147929574), Arg328Trp (rs149836456), and Thr342Met (rs181818400) in the *PNPLA5* gene. Substitutions Gln99His and Phe150Leu (rs147702402) were detected in the subgroup with low LDL-C levels [104].

## 7. Conclusions

Methods for the identification of the aforementioned genes and the examined cohorts are summarized in Table 1.

The molecular genetic diagnosis of FH plays a decisive role when the patients are prescribed modern classes of lipid-lowering agents. In 2018, the Familial Hypercholesterolemia Foundation initiated an assessment of the usefulness of genetic testing for FH and invited an international expert group. They recommended that the genetic testing for FH become a standard of disease management in people with definite or probable FH and for their relatives in the at-risk group [109]. The testing should cover the genes encoding LDLR, apolipoprotein B (APOB), and pro-protein convertase subtilisin/kexin 9 (PCSK9); analysis of other genes may also be necessary depending on a patient’s phenotype. The expected benefits from this implementation of the genetic testing for FH are 1) greater accuracy of diagnosis, 2) higher effectiveness of cascade testing of the patients’ relatives, 3) initiation of treatment at a younger age, and 4) more precise stratification of cardiovascular risk [109]. Among the patients with a high level of LDL-C and a confirmed mutation in one of the above genes associated with FH, there is a 22-fold higher risk of cardiovascular disease [110]. Timely diagnosis allows not only to decrease the risk of cardiovascular diseases in the proband, but also to examine the proband’s relatives for the purpose of diagnosis and primary prevention of cardiovascular diseases.

In 2019, Luis Masana and co-workers proposed a new classification of FH on behalf of the Expert Group of the Spanish Arteriosclerosis Society (Table 2). The factors that prompted the creation of the new classification were 1) discrepancies between the clinical and genetic diagnosis of FH, when FH-associated mutations are not found in people with the clinical diagnosis of definite FH; 2) variation in the clinical signs of FH; 3) high risk of vascular complications among patients with monogenic or polygenic FH; and 4) treatment of FH confirmed by molecular genetic methods with new classes of drugs [111].

Molecular genetic testing of additional genes, including *STAP1*, *CYP7A1*, *LIPA*, *ABCG5*, *ABCG8*, and *PNPLA5*, may be useful for the early diagnosis of polygenic FH, i.e., patients with clinically confirmed (definite) FH, but without the major mutations associated with FH (to be distinguished from nonfamilial multifactorial hypercholesterolemia). This approach may also be helpful for the early diagnosis of FH combined with hypertriglyceridemia: a subgroup of FH patients with combined hyperlipidemia fulfilling the criteria of clinically definite FH with comorbid hypertriglyceridemia. The genes *CYP7A1* and *PNPLA5* can be added to the panel for diagnosing FH after additional studies are conducted on groups of mutation-negative patients with definite FH.

There is little consensus among laboratories on which other genes should be included in the genetic testing. The research on genes whose variants modify the FH phenotype is a separate interesting topic. For example, it has been shown that lipoprotein A is associated with refractory hyperlipidemia in patients with FH (https://www.ncbi.nlm.nih.gov/pubmed/31663632). Additionally, mutations of the *LPA* gene can be the cause of a polygenic form of hyperlipidemia. Langsted et al. (2016) suppose that this gene is a modifier of the FH phenotype. In patients with a confirmed FH diagnosis, the analysis of common variants and rare mutations in the *LPA* gene may be useful for the prediction of complications (long-term prognosis) [112]. Other genes *APOE* (apolipoprotein E), *NPC1L1* (NPC1-like intracellular cholesterol transporter 1), *SORT1* (sortilin 1), *MYLIP* (myosin regulatory light chain interacting protein), *INSIG2* (insulin induced gene 2), *TM6SF2* (transmembrane 6 superfamily member 2), *LIMA1* (LIM domain and actin binding 1), and *CCDC93* (coiled-coil domain containing 93) also hold promise for research on their possible association with FH. The research on the genes whose variants modify the FH phenotype is a separate interesting topic for future review and investigation.

The screening of humans for *APOE* mutation is warranted for molecular diagnostics if the phenotype of FH is combined with hypertriglyceridemia and dysbetalipoproteinemia along with mutations of *LDLR*, *APOB*, and *PCSK9* [113]. The strongest evidence of an association with hyperlipidemia has been found for Leu167del of apolipoprotein E [114,115].

The protein encoded by the *NPC1L1* gene takes up free cholesterol into cells through vesicular endocytosis and plays a critical role in the absorption of intestinal cholesterol (https://www.ncbi.nlm.nih.gov/gene/29881). Polymorphic variations in this gene are associated with plasma total cholesterol and LDL-C levels and coronary heart disease risk [116]. *NPC1L1* promoter variants may partially explain the hypercholesterolemic phenotype of some subjects with FH without mutations in *LDLR*, *APOB*, and *PCSK9* [117,118].

The product of the *SORT1* gene participates in the regulation of trafficking of various proteins to either the cell surface or subcellular compartments, such as lysosomes and endosomes. The expression levels of this gene may influence the risk of myocardial infarction (https://www.ncbi.nlm.nih.gov/gene/6272). A total of 15 nonsynonymous substitutions in *SORT1* have been investigated in FH patients. Even though sortilin (encoded by this gene) binds and internalizes LDL through receptor-mediated endocytosis, mutations in *SORT1* are unlikely to cause autosomal dominant hypercholesterolemia and may have only a marginal effect on plasma LDL-C levels [119].

Genome-wide association studies have shown that sequence variants at the *MYLIP* locus are associated with variations of LDL-C concentration in humans [120]. The polymorphisms rs12464355 and rs7566605 of the *INSIG2* gene have been implicated in hypercholesterolemia and aberrations of serum lipid parameters, especially LDL-C levels. The rs10490626 polymorphism correlates with total cholesterol and LDL-C levels according to a genome-wide association study and meta-analysis [120]. The rs58542926 polymorphic variant of the *TM6SF2* gene is used to identify individuals with a higher susceptibility to chronic liver diseases, especially hepatocellular carcinoma, cirrhosis, alcoholic liver disease, and nonalcoholic fatty liver disease [121,122]. *LIMA1* encodes a negative regulator of cholesterol absorption, and its mutations lead to a decrease in the LIMA1 protein level [123]. In studies on mice and humans, LIMA1 has been identified as a key protein regulating intestinal cholesterol absorption [124]. The CCDC93 protein is reported to be a part of the COMMD–CCDC22–CCDC93 (CCC) complex, which is involved in a novel regulatory mechanism behind intracellular trafficking of LDLR [125,126]. CCDC93 takes part in LDLR recycling and LDL uptake in the liver [127]. A common variant in *CCDC93*, p.Pro228Leu (rs17512204), is associated with enhanced functioning of the CCC complex, which is an endosomal sorting engine that orchestrates LDLR recycling [128].

Thus, genes *APOE*, *NPC1L1*, *SORT1*, *MYLIP*, *INSIG2*, *TM6SF2*, *LIMA1*, and *CCDC93* are related to LDL-C concentration, regulation of the lipid metabolism, or atherosclerosis, but their association with the FH phenotype is not proven yet.

The use of molecular genetic diagnostics of FH in clinical practice allows clinicians to identify the disease at early stages—before the complications of progressive atherosclerosis—and to prescribe an adequate lipid-lowering therapy [129]. All the families in which the FH-associated mutations are detected should be specifically examined by the method of cascade genetic screening for the identification of new FH cases. Regardless of genetic testing, all families with FH require constant observation and a specific clinical examination for the identification of relatives that may be at risk of this hereditary disease.

## Figures and Tables

**Table 1:** Candidate genes of FH and methods of their identification.

Gene	Cohort	Method	Association with lipid metabolism or FH	References
*STAP1*	Family with FH	Exome sequencing	Expression of *STAP1* gene is associated with plasma concentration of lipids (total cholesterol, LDL-C, and triglycerides)	[29]
	Patients with FH and acute coronary syndrome	Targeted next-generation sequencing (NGS) of *LDLR*, *APOB*, *PCSK9*, *APOE*, *STAP1*, *LDLRAP1*, and *LIPA*		[31]
	Case report of FH	Exome sequencing		[32]
	Patients aged ≤ 35 years with LDL-C ≥ 3.4 mmol/L	Exome sequencing		[33]
*CYP7A1*	Case report of FH	Denaturing gradient gel electrophoresis	Variants of *CYP7A1* are associated with total cholesterol and LDL-C levels as well as with changes in risk of IHD	[105]
*LIPA*	Mutation-negative patients with FH	Sanger sequencing	Variants of *LIPA* are associated with high levels of total cholesterol, LDL-C, and triglycerides and low concentration of HDL-C. *LIPA* is also associated with IHD, metabolic complications of obesity, and FH	[65]
	Mutation-negative patients with FH	Sanger sequencing		[64]
	Patients with type II dyslipidemia	Sanger sequencing		[106]
*ABCG5/8*	Case report of FH	Targeted NGS	Variants of *ABCG5/8* are associated with sitosterolemia and higher levels of total cholesterol and triglycerides as well as lower levels of HDL-C	[87]
	Mutation-negative patients with FH	Sanger sequencing		[92]
	Case report of FH	Targeted NGS		[107]
	Case/control study of patients with FH	TaqMan genotyping		[91,93,108]
	Case/control study of patients with FH	Meta-analysis		[108]
*PNPLA5*	Individuals with extremely high and extremely low LDL-C from population-based cohorts	Whole-exome sequencing	Variants of *PNPLA5* are associated with extremely high LDL-C levels	[104]

**Table 2:** Clinical classification of patients with FH according to the Expert Group of the Spanish Arteriosclerosis Society, 2019 [107].

1.	Patients with clinically confirmed (definite) FH and a functional mutation in one copy of genes *LDLR*, *ApoB100*, and *PCSK9*
2.	Homozygous FH: both alleles are mutant
3.	Polygenic FH: patients with clinically confirmed FH but without detectable mutations associated with FH (to be distinguished from nonfamilial multifactorial hypercholesterolemia)
4.	FH combined with hypertriglyceridemia: a subgroup of patients with familial combined hyperlipidemia fulfilling the criteria of clinically definite FH with comorbid hypertriglyceridemia

## List of Abbreviations

aa	amino acid residues
FH	familial hypercholesterolemia
HDL-C	high-density lipoprotein cholesterol
IHD	ischemic heart disease
LDL-C	low-density lipoprotein cholesterol
NGS	next-generation sequencing
TC	total cholesterol
TG	triglycerides
